# *O*-GlcNAc Modification of NFκB p65 Inhibits TNF-α-Induced Inflammatory Mediator Expression in Rat Aortic Smooth Muscle Cells

**DOI:** 10.1371/journal.pone.0024021

**Published:** 2011-08-31

**Authors:** Dongqi Xing, Kaizheng Gong, Wenguang Feng, Susan E. Nozell, Yiu-Fai Chen, John C. Chatham, Suzanne Oparil

**Affiliations:** 1 Vascular Biology and Hypertension Program, Division of Cardiovascular Disease, Department of Medicine, University of Alabama at Birmingham, Birmingham, Alabama, United States of America; 2 Department of Cell Biology, University of Alabama at Birmingham, Birmingham, Alabama, United States of America; 3 Division of Molecular and Cellular Pathology, Department of Pathology, University of Alabama at Birmingham, Birmingham, Alabama, United States of America; 4 Division of Cardiovascular Disease, Department of Medicine, Yangzhou University, Yangzhou, Jiangsu, China; Pennington Biomedical Research Center, United States of America

## Abstract

**Background:**

We have shown that glucosamine (GlcN) or *O*-(2-acetamido-2-deoxy-D-glucopyranosylidene)amino-*N*-phenylcarbamate (PUGNAc) treatment augments *O*-linked-N-acetylglucosamine (*O*-GlcNAc) protein modification and attenuates inflammatory mediator expression, leukocyte infiltration and neointima formation in balloon injured rat carotid arteries and have identified the arterial smooth muscle cell (SMC) as the target cell in the injury response. NFκB signaling has been shown to mediate the expression of inflammatory genes and neointima formation in injured arteries. Phosphorylation of the p65 subunit of NFκB is required for the transcriptional activation of NFκB. This study tested the hypothesis that GlcN or PUGNAc treatment protects vascular SMCs against tumor necrosis factor (TNF)-α induced inflammatory stress by enhancing *O*-GlcNAcylation and inhibiting TNF-α induced phosphorylation of NFκB p65, thus inhibiting NFκB signaling.

**Methodology/Principal Findings:**

Quiescent rat aortic SMCs were pretreated with GlcN (5 mM), PUGNAc (10^−4^ M) or vehicle and then stimulated with TNF-α (10 ng/ml). Both treatments inhibited TNF-α-induced expression of chemokines [cytokine-induced neutrophil chemoattractant (CINC)-2β and monocyte chemotactic protein (MCP)-1] and adhesion molecules [vascular cell adhesion molecule (VCAM)-1 and P-Selectin]. Both treatments inhibited TNF-α induced NFκB p65 activation and promoter activity, increased NFκB p65 *O*-GlcNAcylation and inhibited NFκB p65 phosphorylation at Serine 536, thus promoting IκBα binding to NFκB p65.

**Conclusions:**

There is a reciprocal relationship between *O*-GlcNAcylation and phosphorylation of NFκB p65, such that increased NFκB p65 *O*-GlcNAc modification inhibits TNF-α-Induced expression of inflammatory mediators through inhibition of NFκB p65 signaling. These findings provide a mechanistic basis for our previous observations that GlcN and PUGNAc treatments inhibit inflammation and remodeling induced by acute endoluminal arterial injury.

## Introduction

Inflammation plays an important role in the pathogenesis of many forms of vascular disease, including responses to acute vascular injury. Previous studies, including our own, have shown that inflammatory mediator expression and leukocyte infiltration in injured vessels contribute to vascular remodeling after endoluminal injury [1]–[3]. Glucosamine (GlcN) is an amino sugar that can stimulate *O*-GlcNAc modification (*O*-GlcNAcylation) of proteins by increasing flux through the hexosamine biosynthesis pathway, while *O*-(2-acetamido-2-deoxy-d-glucopyranosylidene) amino-N-phenylcarbamate (PUGNAc) augments *O*-GlcNAc levels by inhibiting *O*-GlcNAcase (OGA), which catalyzes the cleavage of *O*-GlcNAc from modified proteins [4]. We have shown that both GlcN and PUGNAc decrease expression of chemokines [cytokine-induced neutrophil chemoattractant (CINC)-2β and monocyte chemotactic protein (MCP)-1] and adhesion molecules [P-selectin and vascular cell adhesion molecule (VCAM)-1], as well as periadventitial infiltration of neutrophils and monocyte/macrophages in the setting of acute arterial injury in the rat, and that chronic GlcN administration inhibits subsequent neointima formation. These anti-inflammatory and vasoprotective effects are associated with increased levels of *O*-GlcNAc modified proteins in injured blood vessels [5].

We have identified vascular smooth muscle cells (SMCs) as critical “first responders” to acute vascular injury and have developed the tumor necrosis factor (TNF)-α stimulated isolated rat aortic smooth muscle cell (RASMC) as in *in vitro* model that expresses the same pattern of inflammatory mediators as the balloon injured artery *in vivo* [6]. Nuclear factor (NF) κB has been shown to be activated in vascular injury [7], [8], as well as in TNF-α treated vascular SMCs, and NFκB activation is critical for the expression of a variety of genes involved in vascular inflammation [9]–[11]. Phosphorylation of the p65 subunit of NFκB is required for the transcriptional activation of NFκB in a number of ways: by stabilizing p65 protein, regulating DNA-binding activity, decreasing the binding of p65 to IκBα and enhancing its transactivation potential [12]–[18].

A reciprocal relationship between *O*-GlcNAcylation and phosphorylation has been described for some proteins, including estrogen receptor (ER)-β [19] and c-myc [20], suggesting that *O*-GlcNAcylation and phosphorylation may modulate each other. This study tested the hypothesis that increasing NFκB p65 *O*-GlcNAc protein modification with GlcN and PUGNAc treatment inhibits TNF-α-induced inflammatory responses in isolated RASMCs by interfering with NFκB signaling.

## Results

**Figure 1:**
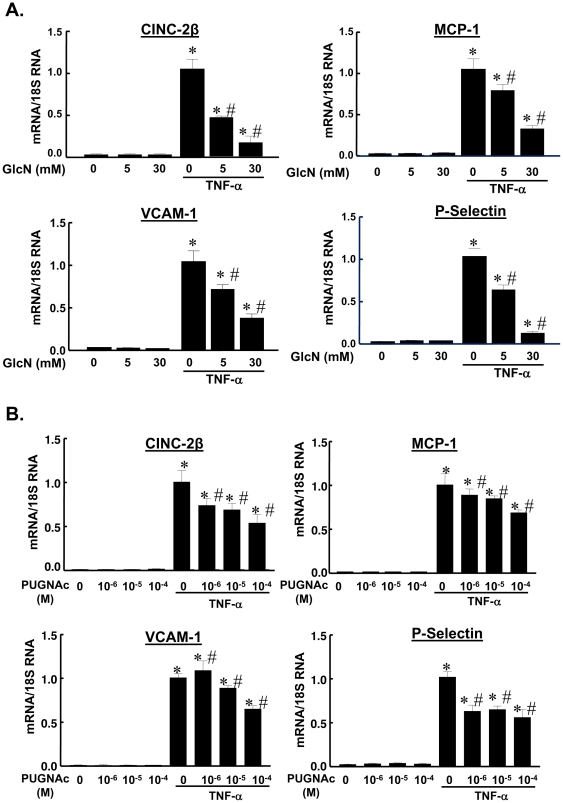
Glucosamine and PUGNAc dose dependently inhibited inflammatory mediator expression in RASMCs. Expression of mRNA for the pro-inflammatory chemokines [cytokine-induced neutrophil chemoattractant (CINC)-2β and monocyte chemotactic protein (MCP)-1] and adhesion molecules [P-selectin and vascular cell adhesion molecule (VCAM)-1] were analyzed by real-time quantitative RT-PCR. Quiescent cells were pretreated with GlcN (5–30 mM), PUGNAc (10^−6^–10^−4^ M) or vehicle for 1 hr, followed by TNF-α (10 ng/mL) for an additional 6 hrs. Data, expressed as means±SEM, are normalized by 18S RNA. Data are standardized to the mean mRNA level of the TNF-α-treated RASMCs. n = 5–6/group. *p<0.05 vs respective vehicle-treated RASMCs; # p<0.05 vs respective TNF-α-treated RASMCs.

To determine whether GlcN and PUGNAc have anti-inflammatory effects in RASMCs in vitro, we examined the effects of GlcN and PUGNAc pretreatment on TNF-α stimulated expression of pro-inflammatory chemokines (CINC-2β and MCP-1) and adhesion molecules [vascular cell adhesion molecule (VCAM)-1 and P-selectin] (Figure 1). Expression of the anti-inflammatory mediator IL-10 was also examined as a negative control. All mediators were expressed at low or undetectable levels in unstimulated vehicle-treated RASMCs. Pretreatment with GlcN or PUGNAc dose-dependently reduced mRNA levels of the chemokines CINC-2β and MCP-1 and the adhesion molecule VCAM-1 in TNF-α treated cells. Both GlcN and PUGNAc also reduced mRNA level of P-selectin, but the PUGNAc effect was not dose dependent over this range. Neither GlcN nor PUGNAc affected TNF-α-induced IL-10 expression (Data not shown). GlcN or PUGNAc alone had no effect on baseline expression of these mediators. These results are consistent with our earlier *in vivo* studies indicating that the VSMC is the target of the anti-inflammatory and vasoprotective effects of GlcN and PUGNAc in acutely injured arteries.

**Figure 2:**
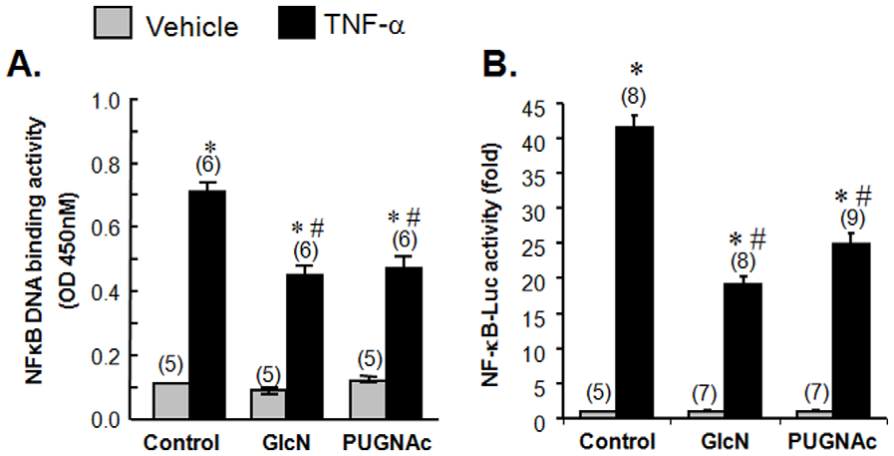
Glucosamine and PUGNAc inhibited NFκB activation in RASMCs. Quiescent cells were pretreated with GlcN (5 mM), PUGNAc (10^−4^ M) or vehicle for 1 hr, followed by TNF-α (10 ng/mL) for an additional 1 hr. A: NFκB p65 DNA binding activity of nuclear extracts was measured using the TransAM NFκB p65 transcription factor assay kit. Data, expressed as mean±SEM, are normalized to protein concentration. B. Cells were transiently co-transfected with pNFκB-Luc, a luciferase reporter plasmid driven by NFκB response elements, and pRL-TK (control for transfection efficiency) using Lipofectamine Plus Transfection Reagent (Invitrogen). Luciferase activities were measured using the dual luciferase assay system. Data, expressed as mean±SEM, are normalized to protein concentration. (n) = number of samples. *p<0.05 vs. vehicle treated RASMCs; # p<0.05 vs. TNF-α-treated RASMCs.

To determine whether the anti-inflammatory effect of GlcN and PUGNAc was mediated through inhibition of TNF-α induced activation of NFκB signaling, we assessed the effects of GlcN and PUGNAc on NFκB p65 DNA binding activity in TNF-α treated cells. TNF-α treatment significantly increased NFκB p65 DNA binding activity compared to vehicle, and this effect was attenuated by pretreatment with GlcN or PUGNAc (Figure 2A). We also found that GlcN and PUGNAc inhibited TNF-α induced NFκB promoter activity, assessed by luciferase activity in cells transfected with the pNFκB-Luc luciferase reporter plasmid (Figure 2B). Cells transfected with the empty control vector showed no lucerfase activity under basal conditions or in response to TNF-α (Data not shown). Taken together, these results indicate that both GlcN and PUGNAc inhibit NFκB p65 activation in TNF-α treated RASMCs.

**Figure 3:**
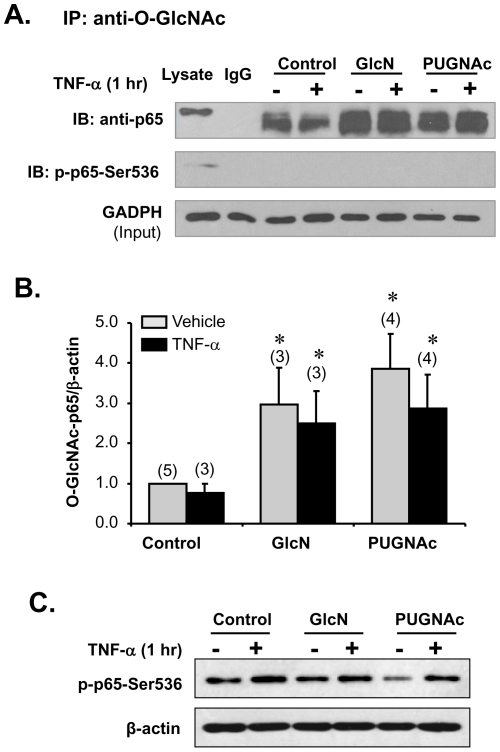
Glucosamine and PUGNAc enhanced NFκB p65 *O*-GlcNAcylation in RASMCs. Quiescent cells were pretreated with GlcN (5 mM), PUGNAc (10^−4^ M) or vehicle for 1 hr, followed by TNF-α (10 ng/mL) for an additional 1 hr. A. *O*-GlcNAc immunoprecipitates (IP) obtained from cellular extracts were analyzed by Western blot for p65 and phos-p65-Ser 536. Cell lysates for IP were probed with GADPH for input loading. B. The band intensity was measured and the ratio of *O*-GlcNAc modified p65 (*O*-GlcNAc-p65) to the corresponding β-actin was calculated. Data, expressed as means±SEM, are *O*-GlcNAc-p65/β-actin ratios standardized to the mean ratio of the vehicle-treated RASMCs shown in bar graph. (n) = number of samples. *p<0.05 vs. vehicle treated RASMCs. C. Representative Western blots of phos-p65-Ser 536 in cell lysate before IP. Blots was reprobed with antibody against β-actin for input loading.

We then tested the hypothesis that GlcN and PUGNAc treatments result in increased *O*-GlcNAcylation of NFκB p65 and attenuated TNF-α-induced phosphorylation of NFκB p65, thereby inhibiting NFκB activation and subsequent expression of NFκB mediated inflammatory genes. To test whether NFκB p65 was *O*-GlcNAcylated, we carried out IP with the anti- *O*-GlcNAc specific antibody 18B10.C7 (Millipore), followed by Western blotting with an anti-p65 specific antibody (Abcam) (Figure 3A, B). We found that GlcN and PUGNAc enhanced *O*-GlcNAcylated p65 levels in cells that had been treated with vehicle or TNF-α; however, TNF-α itself had no effect on p65 *O*-GlcNAc levels in either vehicle treated or GlcN/PUGNAC treated cells. To confirm the specificity of the anti-NFκB p65 antibody, samples were pre-incubated with NFκB p65 blocking peptide and analyzed by Western blot with anti-p65 antibody, and no signal was detected (Figure S1, left panel); reprobing with anti-p65 antibody revealed a strong 65KD band (Figure S1, right panel). Interestingly, after probing the *O*-GlcNAc IP samples with anti-phos-p65 Ser 536 antibody, no signal was detectable (Figure 3A), while the phos-p65 Ser 536 signal was detected in cell lysate before IP of the same samples (Figure 3C), suggesting that *O*-GlcNAcylated p65 cannot be further phosphorylated in response to TNF-α treatment in our cells.

**Figure 4:**
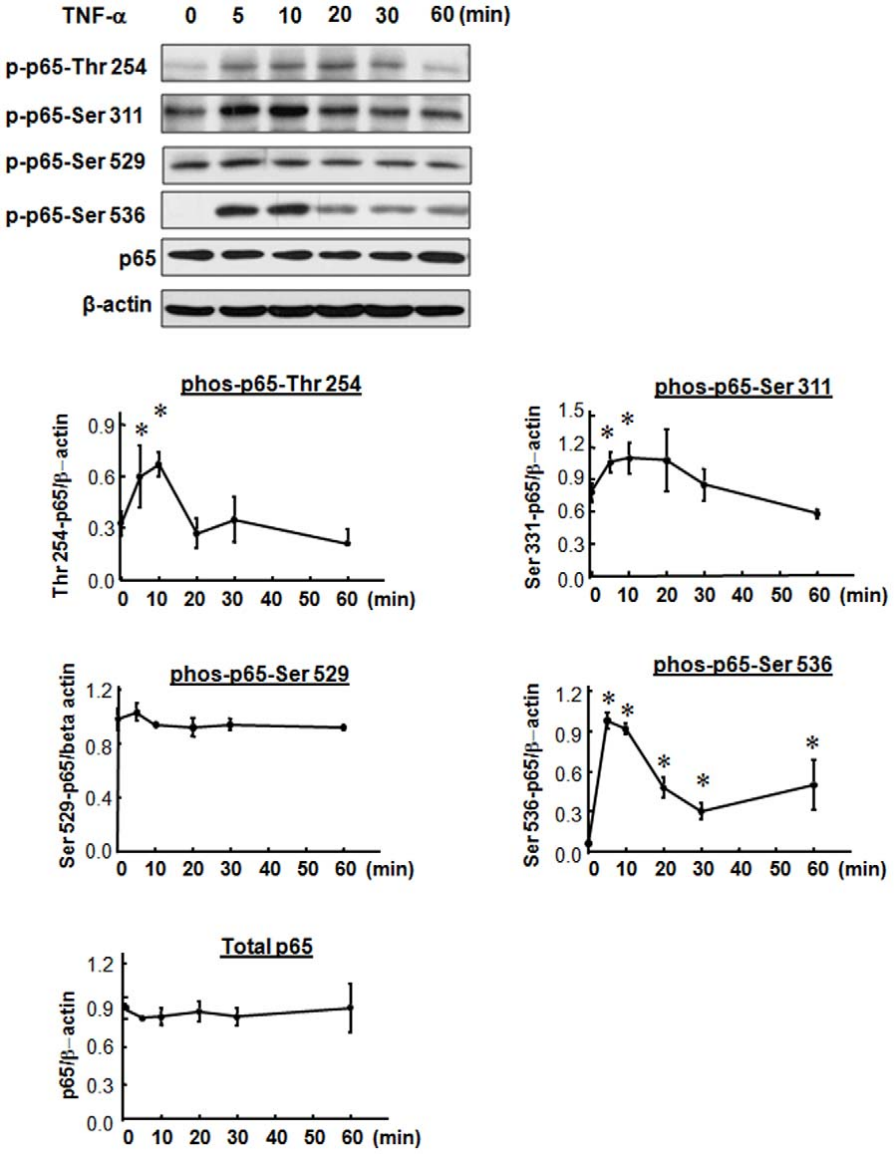
TNF-α phosphorylated NFκB p65 time-dependently at Serine (Ser) and Threonine (Thr) residues in RASMCs. Quiescent cells were treated with TNF-α (10 ng/mL) for the periods indicated. A: Representative Western blots of phosphorylated (phos-) p65 in TNF-α-treated RASMCs with antibody against phos-Thr 254, phos-Ser 311, phos-Ser 529 and phos-Ser 536. Blots were reprobed with antibody against total p65 and β-actin. Data, expressed as mean±SEM, are phos-p65/β-actin ratios as shown in line graphs. n = 3/group. *p<0.05 vs. vehicle treated RASMCs.

Stimulus-induced phosphorylation of multiple amino acid residues in the p65 subunit is required for transcriptional activation of NFκB in various cell types [12]–[18], [21]–[23]. TNF-α has been shown to induce phosphorylation of NFκB p65 at several serine (Ser) and threonine (Thr) residues, i.e. Thr 254, Ser 276 and 311 in the Rel homology domain (RHD) and Ser 529 and 536 in the transactivation domain (TAD) in a variety of cell types. Phosphorylation of these sites has been shown to stabilize p65 protein (Thr 254) [12], regulate DNA-binding activity (Ser 276) [13], decrease the binding affinity of p65 to IκBα (Ser 536) [14] and enhance transactivation potential (Ser 276, 311, 529 and 536) [15]–[18], [22], [23]. We observed very low levels of phos-p65-Thr 254 and phos-p65-Ser 536, and moderate levels of phos-p65-Ser 311 and 529 in whole cell extracts from vehicle-treated RASMCs (Figure 4); however, within 10 min of TNF-α treatment, phosphorylation of p65 at Thr 254 and Ser 311 increased significantly (by 20% and 2-fold, respectively) and then fell to basal levels at 20 or 30 min (Figure 4). Levels of phos-p65-Ser 536 peaked within 10 min of TNF-α stimulation at ∼10-fold baseline, and remained elevated at 60 min (Figure 4). Phosphorylation of p65 at Ser 529 was not altered in response to TNF-α treatment. Phosphorylation of p65 at Ser 276 was not detected in either vehicle or TNF-α treated cells (Data not shown).

**Figure 5:**
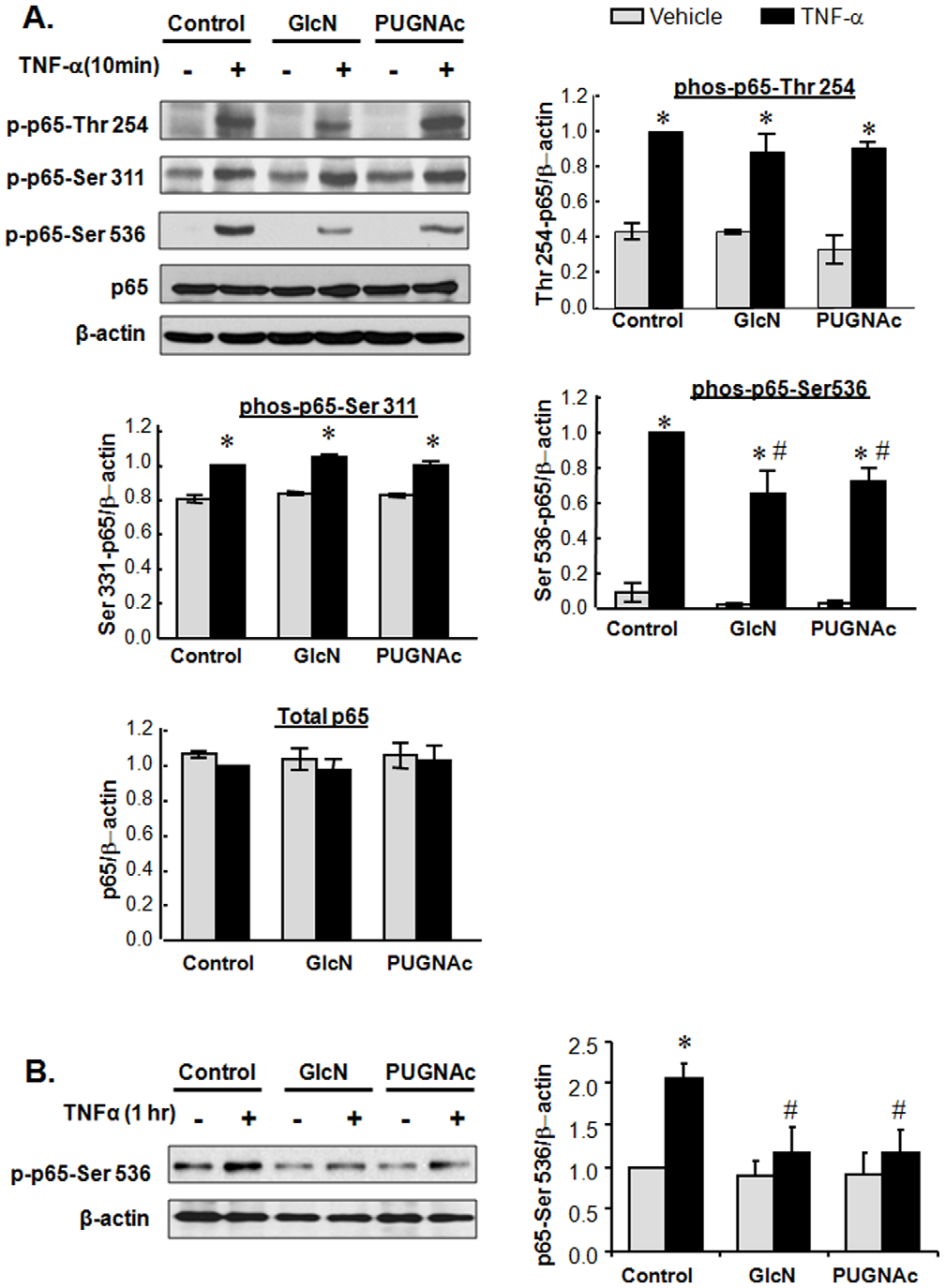
Glucosamine and PUGNAc inhibited phosphorylation of NFκB p65 at Serine 536 in RASMCs. Quiescent cells were pretreated with GlcN (5 mM), PUGNAc (10^−4^ M) or vehicle for 1 hr, followed by TNF-α (10 ng/mL) for an additional 10 min (A) or 1 hr (B). A: Representative Western blots of phos-Thr 254, phos-Ser 311 and phos-Ser 536, in vehicle, GlcN or PUGNAc±TNF-α treated RASMCs. Blots were reprobed with antibody against total p65 and β-actin. B: Representative Western blots of phos-Ser 536 in vehicle, GlcN or PUGNAc±TNF-α treated RASMCs. Blots were reprobed with antibody against β-actin. Data, expressed as mean±SEM, are phos-p65/β-actin ratios as shown in the bar graphs. n = 3–6/group. *p<0.05 vs. vehicle treated RASMCs; # *p*<0.05 vs. TNF-α-treated RASMCs.

Treatment with GlcN or PUGNAc alone had no effect on levels of phos-p65 or total p65 compared to vehicle; however, both GlcN and PUGNAc significantly attenuated TNF-α–induced Ser 536 phosphorylation of NFκB p65 by ∼40% (Figure 5A) at 10 min of treatment, but had no effect on TNF-α–induced phosphorylation of NFκB p65 at Ser 311 or Thr 254. GlcN and PUGNAc also inhibited the late phase of TNF-α-induced phosphorylation of p65 at Ser 536 (Figure 5B). These results suggest that GlcN and PUGNAc primarily affect TNF-α induced phosphorylation of Ser 536 in RASMCs, raising the intriguing possibility that the key *O*-GlcNAc modification sites responsible for the attenuation of NFκB activation are in the vicinity of Ser 536, this means *O*-GlcNAc modification sites are near Ser 536, not necessarily on Ser 536.

**Figure 6:**
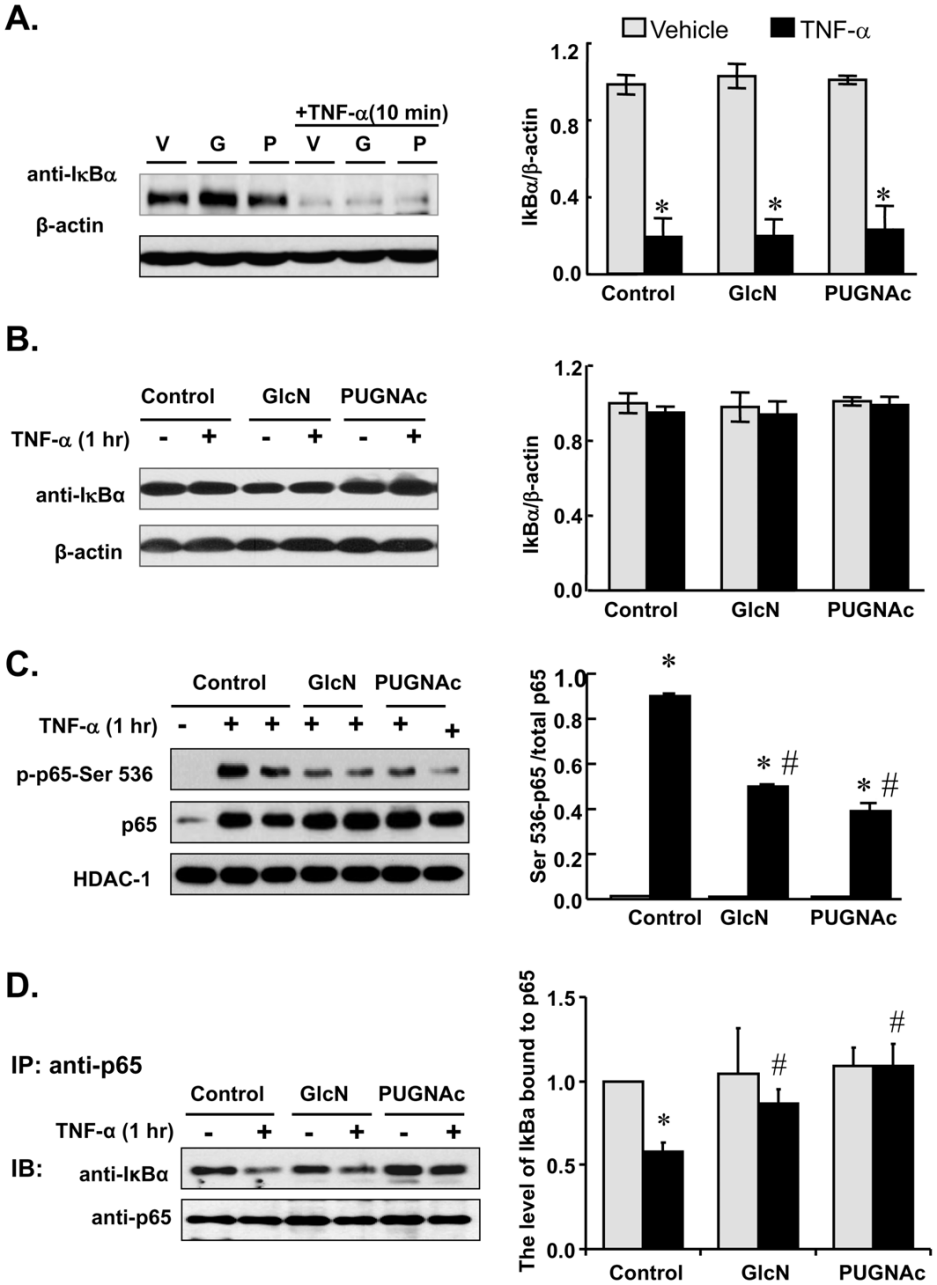
Glucosamine and PUGNAc inhibited nuclear NFκB p65 Serine 536 phosphorylation and promoted the binding of IκBα to NFκB p65 in RASMCs. Quiescent cells were pretreated with GlcN (5 mM), PUGNAc (10^−4^ M) or vehicle for 1 hr, followed by TNF-α (10 ng/mL) for an additional 10 min (A) or 1 hr (B, C, D). A, B: Left: Representative Western blots of total IκBα in vehicle, GlcN or PUGNAc±TNF-α treated RASMCs. Blots were reprobed with antibody against β-actin. Right: The band intensity was measured and the ratio of IκBα to the corresponding total NFκB p65 was calculated. C. Left: Representative Western blots of phos-p65-Ser 536 in nuclear extracts of vehicle, GlcN or PUGNAc±TNF-α treated cells. Blots were reprobed with antibody against total NFκB p65 and histone deacetylase (HDAC)-1 (as loading control). Right: The band intensity was measured and the ratio of phos-p65-Ser 536 to the corresponding total NFκB p65 was calculated. D. Left: NFκB p65 immunoprecipitates (IP) obtained from cellular extracts were analyzed by Western blot for IκBα and NFκB p65 (as input control). Right: The band intensity was measured and the ratio of IκBα to the corresponding NFκB p65 was calculated. Data were expressed as means±SEM. n = 3 or 6/group; *p<0.05 vs. vehicle treated RASMCs; # *p*<0.05 vs. TNF-α-treated RASMCs.

IκBα functions as a key negative regulator of NFκB activity, acting in both the cytoplasm and nucleus. Thus, alterations in IκBα could contribute to the inhibitory effects seen with GlcN and PUGNAc treatment. However, GlcN and PUGNAc had no effect on cytoplasmic degradation or resynthesis of IκBα in response to TNF-α treatment in RASMCs (Figure 6A, B). We also tested the possibility that increasing *O*-GlcNAcylation of p65 altered the basal nucleocytoplasmic shuttling properties of the NFκB-IκBα complex, favoring nuclear export of NFκB. We observed no detectable phos-p65-Ser 536 and very low levels of total p65 in nuclear extracts from vehicle-treated cells (Figure 6C). In response to TNF-α, phos-p65-Ser 536 and total p65 increased significantly in this nuclear preparation. Pretreatment with GlcN or PUGNAc significantly reduced the level of phos-p65-Ser 536 without affecting total p65 in nuclear extracts of TNF-α treated cells. Treatment with GlcN or PUGNAc alone had no effect on nuclear levels of phos-p65-Ser 536 or total p65 compared to vehicle (Data not shown). These results are consistent with the findings in the whole cell lysates, indicating that GlcN and PUGNAc inhibit phosphorylation of p65 at Ser 536, but not nuclear retension of total p65.

To determine whether GlcN and PUGNAc enhanced the interaction of IκBα and NFκB p65, RASMCs were treated with TNF-α or vehicle for 1 hr and subjected to IP with anti-NFκB p65 followed by immunoblotting (IB) with anti-IκBα. The amount of IκBα that coimmunoprecipitated with NFκB p65 decreased dramatically in response to TNF-α; this was prevented by pretreatment with GlcN or PUGNAc (Figure 6D). These results suggest that GlcN and PUGNAc treatment enhanced the binding of p65 to IκBα without altering protein levels of IκBα or NFκB p65 in cells treated with TNF-α.

## Discussion

This study provides the first demonstration that GlcN or PUGNAc treatment inhibits TNF-α induced inflammatory responses in SMCs by inhibiting NFκB p65 Ser 536 phosphorylation, thus increasing sequestration of NFκB p65 by IκBα and thereby attenuating activation of NFκB signalling. These provocative findings provide a mechanistic basis for our previous *in vivo* observations that GlcN and PUGNAc decrease expression of inflammatory mediators and infiltration of neutrophils and monocytes into the periadventitial region of balloon injured rat carotid arteries at a very early time point following endoluminal injury, ultimately resulting in attenuation of neointima formation and adverse vascular remodeling [5].

*O*-GlcNAcylation is an important regulatory mechanism that modulates stress responses in the cardiovascular system [24]–[36]. For example, increasing *O*-GlcNAc levels in cardiac myocytes by treatment with GlcN, PUGNAc or NAG-Bt and NAG-Ae, highly selective *O*-GlcNAcase inhibitors, protects against ischemia-reperfusion injury [24]–[29] and ischemic injury related to myocardial infarction [30]. PUGNAc and GlcN treatments also result in improved cardiac function and organ perfusion and reduced circulating levels of IL-6 and TNF-α in association with increased *O*-GlcNAc protein modification in the heart, liver, and kidney of rats subjected to trauma-hemorrhage [31]–[33]. In this model, attenuation of inflammatory mediator expression is related to reduce NFκB activation [33]. These findings led us to postulate that the anti-inflammatory effects of GlcN and PUGNAc treatment that we had observed in the injured rat carotid artery are related to *O*-GlcNAcylation of components of the NFκB signaling pathway, resulting in reduced NFκB activation and an attenuated vascular injury response. This hypothesis is consistent with previous observations that activation of the NFκB pathway plays an important role in the response to acute vascular injury in animal models [7]–[8], and that blockade of NFκB by a variety of means, including antisense p65, an NFκB decoy or by overexpression of IκBα, effectively inhibits inflammatory responses in injured arteries [37]–[39].

Using the TNF-α treated RASMC, we have demonstrated that increasing NFκB p65 *O*-GlcNAc modification with GlcN or PUGNAc treatment inhibits NFκB activation and have delineated the molecular mechanisms of this effect. We have shown that the p65 subunit of NFκB is a target for *O*-GlcNAc modification in GlcN or PUGNAc treated cells, and that this post translational modification prevents its phosphorylation in response to TNF-α, suggesting a reciprocal relationship between *O*-GlcNAcylation and phosphorylation of NFκB p65 in RASMCs. We further showed that, in cells pretreated with GlcN or PUGNAc, levels of *O*-GlcNAcylated NFκB p65 were increased, and phosphorylation of Ser-536 on NFκB p65 was significantly reduced in response to TNF-α. Ser-536 is located in the COOH terminal TAD of p65 and its phosphorylation plays a key role in transcriptional activation in response to stimuli such as TNF-α [18], [23], [40]. Consistent with our findings in whole cell lysates, TNF-α-induced phosphorylation of Ser-536 on p65 in nuclear extracts was also reduced in cells pretreated with GlcN or PUGNAc. As a consequence, overall NFκB p65 activation (determined by TransAM NFκB p65 transcription factor assay and NFκB luciferase reporter assay) and the p65 mediated expression of inflammatory genes were significantly reduced.

Our demonstration of anti-inflammatory/vasoprotective effects of GlcN in VSMCs and injured arteries is consistent with a large body of literature showing that GlcN inhibits inflammation and related pathological processes in noncardiovascular cells and tissues. GlcN has been shown to suppress expression of the proinflammatory mediators IL-6 and cyclooxygenase-2 in human chondrocytes [41], [42], to inhibit NFκB activation and IL-1β bioactivity in rat chondrocytes [43], to downregulate TNF-α-induced expression of ICAM in human retinal pigment epithelial cells [44], to suppress neutrophil functions such as superoxide generation, phagocytosis, granule enzyme release, and chemotaxis [45], and to inhibit CD3-induced T cell activation [46]. Our findings identifying NFκB p65 as a target for *O*-GlcNAc modification and exploring the inhibitory effect of this post-translational modification on NFκB activity provide a mechanistic basis for the above observations.

Our finding of an apparent reciprocal relationship between *O*-GlcNAcylation and phosphorylation of NFκB p65 in RASMCs is consistent with a large body of evidence that *O*-GlcNAcylation is akin to phosphorylation in that it occurs on serine and/or threonine side chains of proteins, cycles rapidly upon cellular activation, and may interfere with signaling pathways that are mediated by phosphorylation [47]. *O*-GlcNAc and phosphate can either competitively occupy a single site or proximal sites, or noncompetitively occupy different sites on a substrate. Some proteins such as p53 and murine estrogen receptor β are reciprocally modified by *O*-GlcNAc and phosphate, and this reciprocal relationship has been shown to be functionally significant [19], [20], [48].

Our data demonstrate that increasing NFκB p65 *O*-GlcNAc modification with GlcN or PUGNAc treatment prevents TNF-α-induced phosphorylation of p65 at Ser 536, but not phosphorylation of p65 at Thr 254 and Ser 311, indicating that the specific amino acid residues of NFκB p65 that can be *O*-GlcNAcylated in GlcN or PUGNAc treated RASMCs are in the vicinity of Ser 536. These results contrast with previous observations of increased NFκB activity in association with increased *O*-GlcNAc modification of Thr 352 in NFκB p65 in RASMCs under hyperglycemic conditions [49], [50]. In our study, *O*-GlcNAc modification of NFκB p65 was a direct result of GlcN or PUGNAc treatment without the confounding effect of high glucose, which can lead to oxidative stress and advanced glycation end-product accumulation, as in diabetic models [51]. Further, the finding of different sites of phosphorylation/*O*-GlcNAc modification (Ser 536 in our study, Thr 352 in the previous study) may account for differential effects on NFκB activity and downstream inflammatory events.

We are aware that due to the lack of selective antibody for *O*-GlcNAc p65, in the IP experiments we performed, an *O*-GlcNAc modified protein that is bound to p65, rather than *O*-GlcNAc p65 per se might account for our results. Accordingly, our results need to be confirmed by other approaches in the future. Another limitation of the current study is the lack of specificity of the pharmacologic agents used to elicit increases in protein *O*-GlcNAcylation in our cells. We are aware that in addition to increasing *O*-GlcNAc levels, GlcN also increases UDP-GlcNAc, which is used for multiple N-glycosylation reactions that are involved in protein synthesis. Glucosamine-6-phosphate could potentially be metabolized to fructose-6-phosphate, thereby increasing glycolytic flux. Thus, the anti-inflammatory effect seen here with GlcN treatment could potentially be mediated via a number of other pathways. PUGNAc is a potent competitive inhibitor of OGA, but also inhibits other glycoside hydrolases such as the lysosomal β-hexosaminidases and β-N-acetylglucosaminidases [52]. However, data from our group and others have shown that enhancing *O*-GlcNAc modification of proteins using PUGNAc, NAG-Bt or NAG-Ae has anti-inflammatory and cardiovascular protective effects in a range of in vitro and in vivo models [24]–[36]. Further, it has been shown that overexpression of *O*-GlcNAc transferase (OGT) or knockdown of OGA has same effect as PUGNAc in reducing hypoxia-mediated oxidative stress and Ca^2+^ overload in cardiomyocytes [34], [35], while overexpression of OGT has same effect as GlcN in inhibiting LPS-induced activation of NFκB and production of intercellular adhesion molecule-1 and TNF-α in this cell type [33]. Together, this evidence strongly supports the interpretation that the anti-inflammatory effects of GlcN and PUGNAc seen in the current study are indeed mediated via increased *O*-GlcNAc levels.

## Methods

To assess the effect of GlcN and PUGNAc on the TNF-α-induced inflammatory mediator expression, quiescent RASMCs were pretreated with GlcN (5–30 mM), PUGNAc (10^−6^–10^−4^ M) or vehicle and then stimulated with TNF-α (10 ng/ml) for 6 hrs. Inflammatory mediator mRNA expression was assessed by real-time RT-PCR as previously described [2], [5], [6]. To assess the effect of GlcN and PUGNAc on the TNF-α-induced NFκB signaling pathway, quiescent RASMCs were pretreated with GlcN (5 mM), PUGNAc (10^−4^ M) or vehicle and then stimulated with TNF-α (10 ng/ml) for 5, 10, 20, 30, 60 mins or 24 hrs, as described in Results and Figure Legends. Overall NFκB p65 activation was determined by TransAM NFκB p65 transcription factor assay and NFκB luciferase reporter assay. NFκB signaling pathway molecules, including IκBα, NFκB p65 and phosphorylated NFκB p65 (phos-p65) at different amino acid residues (Threonine 254, Serine 276, 311,468, 529 and 536) were measured by Western blot. Binding of IκBα to NFκB p65 and *O*-GlcNAc modified NFκB p65 was assessed using immunoprecipitation (IP), followed by Western blot.

### Cell culture

Primary cultures of RASMCs were derived from 10-week-old female Sprague-Dawley rats (Charles River), as previously described [6]. All protocols were approved by the Institutional Animal Care and Use Committee of the University of Alabama at Birmingham and were consistent with the Public Health Service Policy on Humane Care and Use of Laboratory Animals (Office of Laboratory Animal Welfare, August 2002) and the Guide for the Care and Use of Laboratory Animals published by National Institutes of Health (NIH Publication No. 96-01, revised in 2002). The animal protocol number is 100908574. Cells were cultured in complete medium containing phenol red–free DMEM (Gibco) supplemented with 10% (vol/vol) FBS, 4 mmol/L L-glutamine, 100 U/mL penicillin, and 100 µg/ml streptomycin. RASMCs were used within 5 passages and were identified as RASMCs by their characteristic morphology and positive immunostaining for α-smooth muscle actin (α-SMA, clone 1A4, DAKO). RASMCs were pre-treated with GlcN (5 mM), PUGNAc (10^−4^ M) or vehicle for 1 hr, then incubated with TNF-α (10 ng/mL) for various time periods from 1 to 24 hrs.

### Real-time quantitative RT-PCR analyses of inflammatory mediators

Real-time quantitative RT-PCR analysis was performed as previously described [2], [5], [6]. Total RNA was extracted from cells using TRIzol (Invitrogen, Carlsbad, CA), and treated with DNAase I to remove genomic DNA. The protein- and DNA-free RNA was reverse transcribed to cDNA and analyzed using the SYBR Green RT-PCR kit (Applied Biosystems, Foster City, CA) and specific primers for inflammatory mediators as described before [2], [5], [6]. cDNA was amplified by PCR in the iCycler for 40 cycles and relative RNA levels were calculated using the iCycler software and a standard equation (Applied Biosystems, Foster City, CA). Unknowns were normalized to 18S rRNA and then standardized to the mRNA level of vehicle treated RASMCs.

### NFκB DNA binding activity

Nuclear protein was isolated from RASMCs using the Nuclear and Cytoplasmic Extraction reagents (NE-PER) kit (Pierce). NFκB DNA binding activity in nuclei was determined using the TransAM NFκB p65 transcription factor assay kit according to the manufacturer's instructions (Active Motif, Carlsbad, CA) [33].

### NFκB luciferase reporter assay

RASMCs were transiently co-transfected with pNFκB-Luc, a luciferase reporter plasmid driven by NFκB response elements (Panomics), and pRL-TK, a thymidine kinase promoter-Renilla luciferase reporter plasmid (as a control for transfection efficiency) using Lipofectamine Plus Transfection Reagent (Invitrogen). Luciferase activities were measured by using the Dual Luciferase Assay System (Promega, Madison, WI) with a luminometer and normalized to protein concentration.

### Western Blot Analyses

*O*-GlcNAc, IκBα, NFκB p65 and phosphorylated NFκB p65 (p-p65) in RASMCs were determined by Western blot analysis using anti- *O*-GlcNAc antibody CTD110.6, anti-IκBα (Santa Cruz), NFκB p65 (Abcam), and phos-p65 antibodies (Cell Signaling) [5]. Protein loading was assessed by stripping the membranes and reprobing with anti-β-actin antibody or histone deacetylase (HDAC)-1 (Sigma).

### Co-immunoprecipitation (Co-IP) analysis

RASMCs were lysed in Co-IP buffer (120 mM NaCl, 20 mM Tris, pH 7.5, 2 mM EDTA, 1% Triton-X100, 1 mM sodium vanadate, 10% glycerol) containing 0.5 mM PMSF and 20 µg/ml apotinin, and then centrifuged at 12,000 g for 15 min at 4°C. Protein concentration was determined by a Bradford-based method (Bio-Rad). Following cell lysis, 600 µg of total protein per sample were precleaned with normal rat/mouse IgG and proteinA/G-plus beads (Santa Cruz) and then immunoprecipitated with anti- *O*-GlcNAc (Clone 18B10.C7, Millipore) or anti-NFκB p65 antibody (Abcam), respectively, at 4°C for overnight. The bound proteins on proteinA/G-plus beads were washed using Co-IP buffer, centrifuged, eluted with 2X sample loading buffer, boiled at 95°C for 5 min and stored at −80°C. Each Co-IP experiment was repeated at least three times.

### Statistical Analysis

Data are expressed as mean±SEM. Statistical analysis was performed with one-way ANOVA or Student's t test, as appropriate. Values of P<0.05 were considered significant.

## Supporting Information

Figure S1 **Specificity of our anti-NFκB p65.** *O*-GlcNAc IP obtained from cellular extracts was analyzed by Western blot with anti-p65 antibody pre-incubated with blocking peptide and no signal was detected (left). The blot was then reprobed with anti-NFκB p65 antibody and intense 65KD bands were detected (right).
(TIF)
