# Supplementary figures and images for: O-GlcNAc Modification of NFκB p65 Inhibits TNF-α-Induced Inflammatory Mediator Expression in Rat Aortic Smooth Muscle Cells
Source: PLoS One. 2011 Aug 31;6(8):e24021. doi: 10.1371/journal.pone.0024021 (PMC3164132; doi:10.1371/journal.pone.0024021)

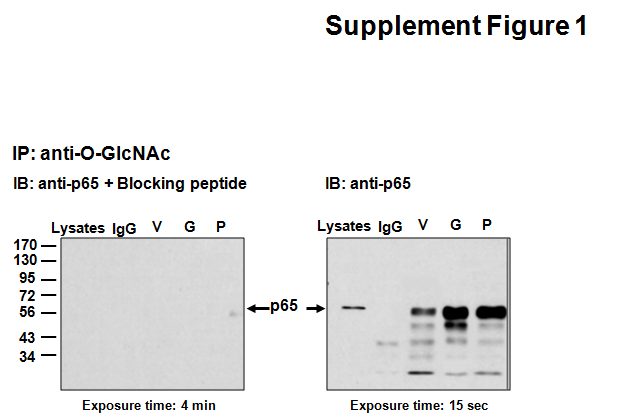

Supplement: Figure S1 — Specificity of our anti-NFκB p65. O-GlcNAc IP obtained from cellular extracts was analyzed by Western blot with anti-p65 antibody pre-incubated with blocking peptide and no signal was detected (left). The blot was then reprobed with anti-NFκB p65 antibody and intense 65KD bands were detected (right). (TIF) [file pone.0024021.s001.tif]
